# A new device and method for detecting the permeability of articular cartilage

**DOI:** 10.3389/fbioe.2026.1807678

**Published:** 2026-03-19

**Authors:** Fuyang Cao, Haibin Deng, Guohao Zhang, Ningning Ma, Binghang Zhang, Xianda Che, Gaige Wu, Pengcui Li, Li Guo, Xiaochun Wei

**Affiliations:** 1 Department of Orthopedics, Second Hospital of Shanxi Medical University, Taiyuan, China; 2 Shanxi Key Laboratory of Bone and Soft Tissue Injury Repair, Taiyuan, China

**Keywords:** articular cartilage, convective principle, Darcy’s law, mechanical loading, permeability testing

## Abstract

**Objective:**

Articular cartilage relies on synovial fluid for substance exchange, with permeability being a key parameter linked to its physiological functions and degenerative disease mechanisms. Existing detection techniques are limited by detachment from physiological mechanical environments, insufficient quantification accuracy, and poor sealing. This study aimed to develop an accurate, reliable, and physiologically relevant device and method for cartilage permeability detection.

**Methods:**

Based on intra-articular convective transport and Darcy’s Law, a modular testing device (permeation, pressure supply, central control modules) was fabricated to simulate the synovial fluid–cartilage–subchondral bone unit under pressure. Porcine femoral condylar cartilage samples were tested with 4 N, 8 N, 16 N (n = 6, per group) constant pressure (1800 s) using an ElectroForce 3,200 system. Permeability coefficients were calculated via Darcy’s Law-derived formulas. Repeatability (single operator, fixed conditions, n = 6) and reproducibility (multiple operators, unified conditions, n = 6) were verified by CV and ANOVA. Human knee cartilage (n = 6) was used to validate practicality for clinical solutions. Data were analyzed with GraphPad Prism 9.5.

**Results:**

Friction force between the push rod and permeation tube was 2.5 N, with no leakage confirmed by eosin dye. Repeatability tests showed mean permeabilities of 1.50 × 10^−17^ m^2^, 1.05 × 10^−17^ m^2^ and 0.67 × 10^−17^ m^2^ at 4 N, 8 N, 16 N (CV ≤ 6.4%). Reproducibility tests yielded a mean permeability of 1.05 × 10^−17^ m^2^ (CV = 7.4%, *P* = 0.9776 > 0.05). Osteoarthritic (OA) cartilage exhibited higher permeability to sodium hyaluronate (0.99 × 10^−17^ m^2^) and interleukin-1β (1.39 × 10^−17^ m^2^) than normal cartilage (0.71 × 10^−17^ m^2^ and 1.39 × 10^−17^ m^2^).

**Conclusion:**

The developed device and method address traditional limitations, featuring excellent physiological relevance, quantification accuracy, and stability. It provides a pivotal tool for studying cartilage physiology, OA pathology, optimizing repair materials and therapeutic strategies, and improving cartilage injury repair evaluation.

## 1 Introduction

As a dense connective tissue covering the articular ends of bones, articular cartilage is primarily composed of chondrocytes and extracellular matrix (ECM) (Carballo et al., 2017). Endowed with its unique viscoelasticity and porous matrix structure, it possesses core functions of excellently cushioning mechanical loads, reducing joint friction, and maintaining motor flexibility, serving as a pivotal tissue for safeguarding the physiological homeostasis of joints (Pueyo Moliner et al., 2025).

Due to its avascular nature, the nutrient supply (e.g., glucose, oxygen) and elimination of metabolic wastes (e.g., lactic acid, carbon dioxide) for chondrocytes completely rely on substance exchange with synovial fluid (Deng et al., 2024). Under physiological conditions, this substance exchange between articular cartilage and synovial fluid follows a “multi-mechanism synergy” mode: Diffusion serves as the basic transport pathway for small-molecule substances (e.g., glucose, oxygen), ensuring the basic metabolic needs of cartilage (DiDomenico et al., 2018); The cartilage deformation pump accelerates the exchange of medium-molecule substances through the “compression-rebound” sponge effect during joint movement (Evans and Quinn, 2006a); Convection, as the core mechanism under joint motion, is driven by hydrostatic pressure in the enclosed intra-articular environment (Garcia et al., 1996). It acts as the decisive force for the transcartyilaginous transport of macromolecular substances (e.g., therapeutic proteins, growth factors) and can significantly enhance transport efficiency compared to simple diffusion (Evans and Quinn, 2006b).

The efficiency of substance exchange between cartilage and synovial fluid directly depends on cartilage permeability—a key parameter characterizing the transport capacity of fluids and solutes across the cartilage matrix (Mansour and Mow, 1976). Permeability not only reflects the structural integrity and microenvironmental homeostasis of cartilage but also directly correlates with the functional indicator of its metabolic efficiency (Zuo et al., 2024). Accurate evaluation of cartilage permeability is of paramount significance for further elucidating the physiological functions of cartilage, revealing the pathological mechanisms of degenerative diseases such as osteoarthritis (OA), optimizing the design of cartilage repair materials, and developing targeted therapeutic strategies.

As one of the key evaluation indicators for cartilage structural integrity, the development of measurement techniques for the cartilage permeability coefficient has garnered sustained attention in the academic community. Currently, mainstream detection technologies can be categorized into two types: One type is static detection methods based on the diffusion mechanism, such as radioactive labeling, fluorescence labeling, and diffusion cell assays (DiDomenico et al., 2018). These methods quantify key parameters (e.g., diffusion coefficient, partition coefficient) by tracking the natural permeation process of solutes in cartilage (Aoki et al., 2012). However, they inherently operate in a static environment without mechanical stimulation, failing to reproduce the convective effects and cartilage deformation pump action induced by cyclic loading during joint movement, thus struggling to reflect the dynamic transport characteristics of fluids and solutes under physiological conditions. The other type is dynamic detection methods integrated with mechanical factors, such as dynamic mechanical analysis (DMA) and microelectromechanical systems (MEMS) sensor-assisted detection (Oldinski et al., 2011; Tang et al., 2009). By directly applying cyclic compressive loads to cartilage, these methods simulate the influence of joint movement on permeability. Nevertheless, they are mostly used for qualitative research and cannot achieve quantification; additionally, they suffer from issues such as complex equipment and high operational difficulty. Furthermore, the accurate quantification of cartilage permeability faces multiple technical challenges: First, the edge sealing problem of irregular cartilage surfaces has not been effectively resolved, requiring experimental devices to have excellent adaptability. Second, traditional permeants cannot be directly detected by equipment and need to be combined with radioactive or fluorescent substances, which not only increases costs but also introduces additional errors (Moore et al., 2016).

Addressing the limitations of existing technologies, such as detachment from the physiological mechanical environment, insufficient quantification accuracy, and poor sealing performance, this study aims to develop a novel device and method for cartilage permeability testing based on the convective principle. The core design features are as follows: (1) Replicating the “synovial fluid–cartilage–subchondral bone” core functional unit. Through a mechanical loading system, the physiological hydrostatic pressure environment (0.1–3 MPa) is accurately reproduced (Lee et al., 2022; Sperber and Wredmark, 1993), significantly enhancing pressure control stability and fully simulating the *in vivo* convective permeation process; (2) Employing displacement sensors with micrometer-level resolution to achieve accurate quantification of permeant volume mediated by cartilage permeability. (3) The core calculation formula based on Darcy’s Law: Darcy’s Law is the fundamental mechanical law that describes the fluid flow through porous media. It is widely applied in fields such as biomedical engineering (e.g., cartilage, bone tissue) and geotechnical engineering. Its core logic is directly related to the material transport mechanism of articular cartilage (Hanshaw et al., 1965; Mow and Torzilli, 1975).

This design resolves issues such as sealing leakage and pressure fluctuations in traditional equipment, enabling precise quantification of permeability differences of cartilage to various solutes (especially macromolecules). By overcoming the limitation of traditional methods being disconnected from physiological scenarios, this technology provides a more physiologically relevant technical tool for further elucidating the substance transport mechanism of articular cartilage, optimizing the design of OA-targeted drugs, and improving the cartilage repair evaluation system.

## 2 Materials and methods

### 2.1 Design principle of the cartilage permeability testing device

As the primary mode of substance exchange between cartilage and synovial fluid within the joint cavity, the convective mechanism plays a crucial role in this process. The main driving force for convection is the hydrostatic pressure exerted by synovial fluid on articular cartilage.

The joint cavity is a closed space: its upper and lower ends are lined with articular cartilage, surrounded by the joint capsule, and filled with synovial fluid. The generation of synovial fluid hydrostatic pressure is the combined effect of the joint cavity’s closed nature and joint movement. The core principle lies in the compression of fluid within a closed space, which generates pressure on the surrounding structures.

To accurately quantify the permeation characteristics of cartilage to synovial fluid under the hydrostatic pressure environment within the joint cavity, it is necessary to first simplify the structure of the complex joint system, construct a reproducible and controllable core functional unit, and then establish the corresponding correlation between the cartilage permeability testing device and the physiological structure based on this unit, laying a foundation for subsequent permeability mechanism research and device application.

**FIGURE 1:**
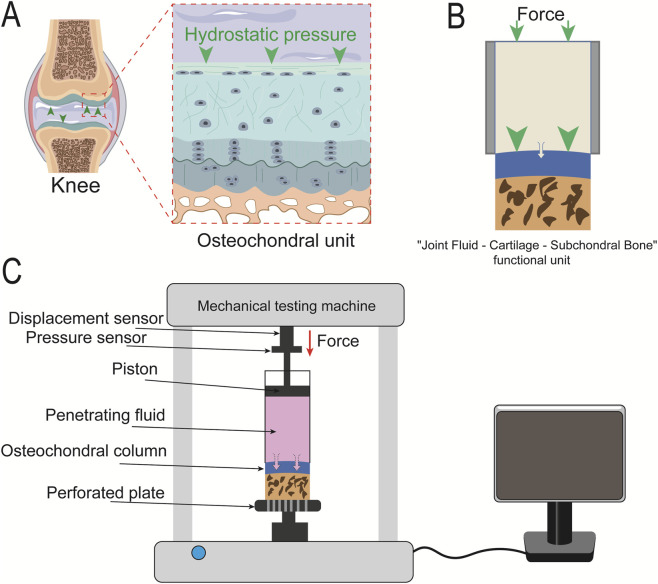
Schematic diagrams of the design principle and overall components of the permeability testing device. **(A)** Schematic diagram of the knee joint structure and the “synovial fluid–cartilage–subchondral bone” core functional unit; **(B)** Schematic diagram of the simplified core functional unit, consisting of an upper layer (synovial fluid/permeant), a middle layer (cartilage), and a lower layer (subchondral bone); **(C)** Overall components of the permeability testing device.

The joint cavity is a complex motion system composed of multiple components such as cartilage, subchondral bone, synovium, and synovial fluid. Among these, the three-layer structure of “synovial fluid–cartilage–subchondral bone” is the core functional unit determining synovial fluid permeation and nutrient transport. The basis for simplification and structural characteristics are as follows:

From a physiological function perspective, synovial fluid, as a solute carrier, penetrates the cartilage matrix to reach chondrocytes under the action of intra-articular hydrostatic pressure (e.g., 0.5–3 MPa during standing and 0.1–0.3 MPa at rest), completing nutrient supply and metabolic waste elimination. As a supporting structure for cartilage, subchondral bone limits the excessive permeation of synovial fluid into deep tissues, forming a closed-loop transport pathway of “synovial fluid permeation–cartilage transport–subchondral bone barrier.” Components with no direct impact on the permeation process, such as the synovium and ligaments, can be excluded (Figure 1A).

Structural simplification was performed based on the aforementioned core functional unit. The design of the cartilage permeability device is as follows:

1. Upper layer (permeant layer): A permeation tube contains the test solution (simulating synovial fluid). By leveraging the incompressibility of the fluid, external mechanical loads are converted into hydrostatic pressure, which acts directly on the cartilage surface, matching the pressure transmission characteristics of synovial fluid *in vivo*.
2. Middle layer (cartilage layer): The key target for permeability testing. Under hydrostatic pressure, the permeant can penetrate the cartilage to a certain extent. Additionally, the natural viscoelastic properties of cartilage are preserved, allowing physiological deformation under hydrostatic pressure to simulate the interaction between cartilage and fluid during the “compression” process *in vivo*.
3. Lower layer (subchondral bone layer): Subchondral bone provides a rigid supportive function, limiting the permeation of the solution into deep tissues. It constructs a unidirectional permeation pathway of “solution→cartilage” and eliminates interference from multi-directional leakage on measurement results (Figure 1B).

The cartilage permeability testing device is based on the core principle of convective transport between synovial fluid and cartilage—which, as a closed space, generates physiological hydrostatic pressure of 0.1–3 MPa (with an average of 1.5 MPa, corresponding to a force of 8 N per unit area) when bony structures compress synovial fluid during daily activities. This pressure drives synovial fluid to permeate the cartilage matrix, and this convective process serves as a key pathway for macromolecular transport, being more consistent with the dynamic mechanical environment *in vivo*.

The permeability testing device is used in conjunction with a mechanical loading instrument integrated with high-precision force sensors and displacement sensors, enabling highly stable mechanical loading. A rigid metal indenter directly contacts the permeant for pressure application, eliminating pressure fluctuations caused by gas compressibility. Meanwhile, the ultra-precision displacement sensor of the mechanical loading instrument performs real-time monitoring of microscale liquid level changes, and these data are recorded in real time by the computer central control unit. This dual high-precision measurement system for pressure difference and displacement ensures excellent accuracy in permeability measurement (Figure 1C).

### 2.2 Design and fabrication of the cartilage permeability testing device

With the core objectives of simulating the mechanical environment of the joint cavity and accurately measuring permeability, the cartilage permeability testing device designed in this study adopts a modular architecture. It mainly comprises three functional modules: a permeation mechanism, a pressure supply mechanism, and a central control system. These modules cooperatively achieve sample fixation, solution pressurization and permeation, data acquisition, and permeability calculation. Among them, the permeation mechanism primarily consists of a permeation tube, a push rod, and a fixing bracket.

#### 2.2.1 Permeation tube

As a key component of the core permeation mechanism, the permeation tube serves multiple functions, including storage of test solutions, transmission of hydrostatic pressure, sealing and fitting of cartilage samples, and constraint of the permeation process. Its structural design and performance parameters directly determine the accuracy and stability of cartilage permeability measurement.

**FIGURE 2:**
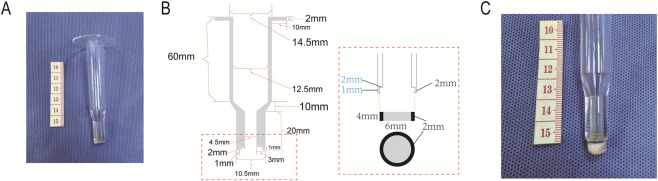
Permeation tube. **(A)** Photograph of the permeation tube; **(B)** Overall structure and dimensional parameters of the permeation tube; **(C)** Direct contact between the rubber gasket and the cartilage surface forming a sealing mechanism.

The overall structure of the permeation tube mainly consists of four parts: the permeation tube main body, top inlet, bottom outlet, and fixing end (Figure 2A). As the basic framework of the permeation tube, the main body is made of quartz glass material with rigidity and transparency. Its inner diameter must match the size of the piston at the compression end of the push rod to ensure uniform pressure application when the push rod moves vertically inside the tube. The tube body needs sufficient strength to withstand the hydrostatic pressure transmitted by the pressure supply mechanism, avoiding deformation under pressure that could affect measurement accuracy.

The top inlet is located at the top of the main tube body, serving as a channel for the push rod to enter and exit. It allows the detachable installation of the push rod (including the compression end piston and push rod body) inside the tube, which applies pressure to the surface of the permeant in the tube through vertical movement.

The bottom outlet is situated at the bottom of the main tube body and acts as a key interface for the permeant to contact the cartilage. Its core design lies in the “integration of a sealing mechanism”: a rubber gasket is installed at the outlet with a height of 4 mm, fixed by a card slot structure (the side wall of the card slot is 2 mm high, and the rubber gasket protrudes 2 mm above the slot) (Figure 2B). This design enables the rubber gasket to directly contact the cartilage surface, forming a tight seal under the pressure of the fixing bracket. It not only prevents leakage of the permeant from the gap between the permeation tube and the cartilage but also avoids tissue damage caused by direct contact between the tube body and the cartilage (Figure 2C).

Fixing end: It is a wing-shaped structure arranged on the outer sidewall of the main tube body. Its core function is to cooperate with the fixing bracket of the permeability device, allowing the permeation tube to be stably fixed on the bracket. Through pressurization, the bottom outlet of the permeation tube is closely attached to the cartilage sample, ensuring the tube body remains vertical during pressure application. This avoids uneven pressure distribution caused by tube inclination, which would compromise the unidirectionality of solution permeation (permeation only along the direction perpendicular to the cartilage surface).

#### 2.2.2 Push rod

As one of the core functional components of the permeation mechanism, the push rod, in conjunction with the permeation tube, achieves three core objectives: hydrostatic pressure application, permeant regulation, and ensuring operational flexibility.

**FIGURE 3:**
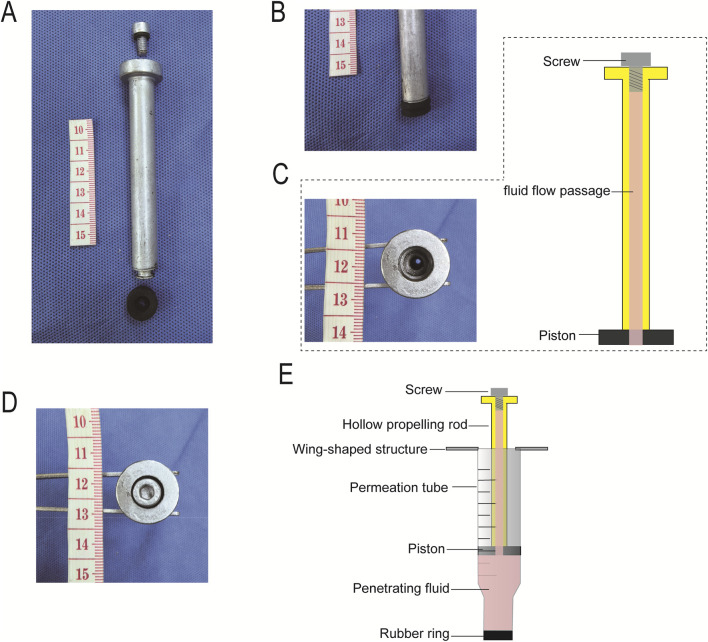
Push rod. **(A)** Photograph of the push rod; **(B)** Compression end and piston of the push rod; **(C)** Fluid channel of the push rod; **(D)** Sealing mechanism and sealing screw of the push rod; **(E)** Fluid channel connected to the permeation tube lumen through the piston opening.

The push rod has an overall rod-like structure, consisting of four parts: the push rod body, compression end, fluid channel, and sealing mechanism (Figure 3A). The compression end is vertically connected to the push rod body and located at its lower end, externally covered with a piston (Figure 3B) that directly contacts the surface of the permeant in the permeation tube. Meanwhile, the piston is in direct contact with the inner wall of the permeation tube, further enhancing the sealing effect and preventing solution leakage from the gap between the compression end and the tube inner wall, ensuring that pressure can be fully applied to the permeant surface. Additionally, the center of the piston is equipped with an opening that connects to the fluid channel.

The fluid channel runs through the center of the push rod body, extending along its length. One end penetrates the end face of the compression end (piston), and the other end extends to the upper end of the push rod body (connected to the sealing mechanism) (Figure 3C). This channel meets the functional requirements of solution injection and air exhaust: it not only facilitates supplementing permeant into the tube after the permeation tube and cartilage are sealed but also expels air from the tube, avoiding pressure transmission errors caused by air compression.

The sealing mechanism is located at the upper end of the push rod body and adopts a detachable sealing screw (Figure 3D) to block the top opening of the fluid channel. It ensures the tightness of the fluid channel after sealing, preventing the permeant in the tube from leaking from the top of the channel under pressure. Meanwhile, it maintains operational convenience, allowing for easy disassembly to replace or supplement the permeant as needed during experiments (Figure 3E).

#### 2.2.3 Fixing bracket

The fixing bracket serves key functions of stably fixing the permeation mechanism, constraining the position of cartilage samples, and adjusting the spatial attitude during the detection process. Its structural design directly determines the fit accuracy between the permeation tube and cartilage, as well as the stability of pressure transmission, making it an important foundation for ensuring the accuracy of permeability measurement.

**FIGURE 4:**
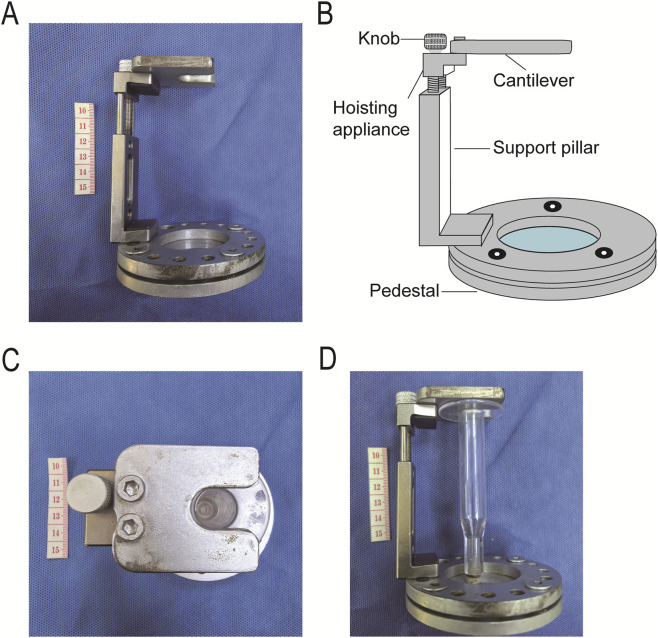
Fixing bracket. **(A)** Photograph of the fixing bracket; **(B)** Component structure of the fixing bracket; **(C)** Cantilever of the fixing bracket; **(D)** Clamping of the permeation tube by the fixing bracket.

The fixing bracket has an overall C-shaped frame structure, achieving the functional integration of “supporting–lifting–clamping” through modular design. It mainly consists of three parts: a fixed base, a lifting mechanism, and a cantilever (Figures 4A,B).

As the basic supporting unit of the fixing bracket, the fixed base is made of high-rigidity stainless steel. Its bottom needs to have sufficient contact area and anti-slip performance to avoid displacement or vibration of the device due to pressure during the detection process.

The lifting mechanism is connected between the fixed base and the cantilever. It is an adjustable mechanism with vertical lifting function, adopting a thread-adjustable elevator. One end of the lifting mechanism is fixed on the fixed base, and the other end is connected to the cantilever (Figure 4B). The vertical position movement of the cantilever is realized through a manual knob, and its lifting accuracy must meet the requirements of fitting and sealing between the permeation tube and the cartilage sample. This ensures that the rubber gasket at the bottom of the permeation tube can be accurately pressed against the surface of the cartilage sample to be tested through fine adjustment, and an appropriate preload is applied to establish the initial sealed state.

The cantilever is installed at the top end of the lifting mechanism. Its shape and size need to match the fixing end (wing-shaped structure) of the permeation tube (Figure 4C) to achieve stable clamping of the permeation tube (Figure 4D).

### 2.3 Operational procedure of the permeation device

#### 2.3.1 Sample preparation

**FIGURE 5:**
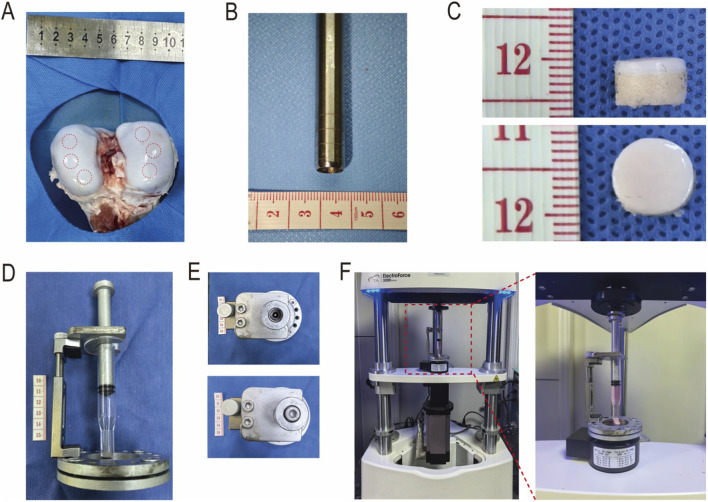
Harvesting of Osteochondral Samples and Installation Procedure of the Permeability Device. **(A)** Femoral condyle of the knee joint from a Landrace pig at a local abattoir and the sampling area of the osteochondral sample; **(B)** Osteochondral trephine with an inner diameter of 8.5 mm; **(C)** Top view and side view of the osteochondral sample; **(D)** Assembly of the permeability testing device; **(E)** Top inlet of the push rod and sealing of the inlet with a sealing screw; **(F)** Permeability device placed between the stage and buffer stage of the mechanical loading instrument.

Porcine osteochondral samples were obtained from adult Landrace pigs (6-month-old, weighing 50–60 kg) at a local abattoir. Within 8 h post-slaughter, the midshafts of the femur and tibia were dissected, and the intact knee joint was retained. The skin, fascia, and joint capsule were sequentially incised layer by layer to expose the knee joint. The anterior and posterior cruciate ligaments as well as the collateral ligaments were transected, and excess muscles and synovial tissue were excised to retain the femoral segment. The femoral condyle cartilage was examined to ensure that its surface was smooth and free of any obvious abnormalities, and only then was the next step of the operation carried out (Figure 5A). A region with a flat cartilage surface was selected, and an osteochondral trephine with an inner diameter of 8.5 × 10^−3^ m was used to harvest osteochondral plugs from the femoral condyle (Figure 5B). The bony segment of the osteochondral plug was trimmed to maintain a sample height of 5 × 10^−3^ m, and the thickness of the cartilage layer (L: m) was recorded (Figure 5C). All samples were stored in phosphate-buffered saline (PBS) at 4 °C for subsequent use.

Human osteochondral samples were obtained from a biobank, derived from the osteochondral tissue resected during total knee arthroplasty (TKA). Human osteochondral samples were harvested from six patients (2 males and four females, aged 58–72 years with a mean of 65.3 ± 4.8 years) who underwent total knee arthroplasty for end-stage knee osteoarthritis; the OA cartilage samples were all classified as Kellgren-Lawrence (K-L) grade IV (severe osteoarthritis) with extensive joint space narrowing, severe osteophyte formation, and subchondral bone sclerosis/cysts, while the relatively normal cartilage samples were obtained from the non-weight-bearing peripheral regions of the same resected specimens with K-L grade I, and were age and gender-matched to the OA cartilage donors. Following the same procedure described above, an osteochondral trephine was used to harvest osteochondral plugs, and the length of the subchondral bone segment was trimmed. The thickness of the cartilage layer was recorded, and all samples were stored in phosphate-buffered saline (PBS) at 4 °C for subsequent use.

#### 2.3.2 Sample installation

The installation procedure is as follows: First, assemble the fixing bracket and raise the lift arm to the highest position. Place the wing-shaped handle of the permeation tube under the cantilever of the fixing bracket, and position the cartilage sample directly below the permeation tube such that the central axes of the permeation tube and the cartilage sample are completely aligned. Slowly rotate the knob of the lift arm to lower the cantilever and permeation tube simultaneously until the rubber gasket at the bottom of the permeation tube is in contact with the cartilage surface. Then lower the cantilever slightly to allow the rubber gasket to compress the cartilage moderately, thereby completely eliminating the gap between the rubber gasket and the cartilage (Figure 5D). Subsequently, insert the piston end of the push rod into the permeation tube to the appropriate position, open the sealing screw at the top, add the permeant until it slightly overflows, and then screw on the sealing screw to achieve sealing (Figure 5E).

Subsequently, the cartilage permeability testing device was placed on the circular stage beneath the mechanical loading instrument (ElectroForce 3,200, United States of America). This instrument has a maximum applied force of 225 N and a maximum stage displacement of 6.5 × 10^−3^ m. The distance between the stage and the circular buffer stage above the mechanical loading instrument was adjusted such that the push rod of the cartilage permeability testing device was in precise contact with the buffer stage (Figure 5F). During pressure application, the stage moves upward toward the buffer stage, exerting pressure on the intermediate test assembly. The buffer stage is connected to a force sensor and a displacement sensor, with a force resolution of 0.01 N and a displacement resolution of 0.01 um, enabling real-time and accurate recording of the upward compressive force and displacement of the stage.

It is important to ensure that when placing the permeability device, the central axes of the permeation tube and osteochondral sample are aligned with the line connecting the centers of the stage and buffer stage. This guarantees that the applied mechanical force is completely perpendicular to the surface of the osteochondral sample, thereby avoiding deviations between the hydrostatic pressure borne by the cartilage and the set value.

#### 2.3.3 Mechanical loading

A constant compressive force was applied to the top of the push rod via compression between the stage and buffer stage of the mechanical loading instrument (ElectroForce 3200, TA Instruments, United States). First, the target force was set, followed by a loading rate of 1 N/s. Upon reaching the target force, this constant load was maintained steadily for 1800 s.

#### 2.3.4 Friction force measurement

A close fit between the permeability tube and the piston assembled at the lower end of the push rod resulted in frictional forces, which could compromise the accuracy of force transmission from the applied load to the osteochondral sample. To quantify this friction, the permeability device was mounted on the mechanical loading instrument (ElectroForce 3200, TA Instruments, United States) without an osteochondral sample installed. A constant displacement profile was set to drive the push rod in a linear motion. During this movement, the computer control unit recorded the displacement and the corresponding driving force in real time. When the push rod advanced at a constant velocity, the recorded force directly represented the frictional force acting between the piston and the inner wall of the permeability tube. In the subsequent experiments, the set pressure loads were all adjusted values obtained by subtracting the frictional force from the actual set values.

#### 2.3.5 Seal integrity testing

Eosin Y dye was added to the permeability tube, followed by continuous loading under the preset pressure for 1800 s. After completion, the osteochondral sample was removed, and the contact area between the sample and the rubber ring of the permeability tube was inspected for red staining. Throughout the loading period, close observation was conducted to check for any dye leakage from the interface between the osteochondral sample and the rubber ring.

#### 2.3.6 Permeation fluids

Standardized distilled water (dynamic viscosity μ = 1 mPa·s), normal saline (μ = 1 mPa·s), 5% glucose solution (μ = 1.2 mPa·s), sodium hyaluronate injection (μ = 27 mPa·s), dexamethasone injection (μ = 1.2 mPa·s), and interleukin-1β (IL-1β) solution (μ = 0.9 mPa·s) were selected as permeation fluids.

### 2.4 Permeability–Darcy’s law

As a porous medium, articular cartilage’s fluid transport properties are typically characterized by Darcy’s law:

$$Q=\frac{k\;*\;A\;*\;\Delta P}{\mu L}$$ (1)

In the formula, Q denotes the volume of permeation fluid flowing through the cartilage sample per unit time (unit: m^3^/s); k represents the permeability coefficient of the cartilage (unit: m^2^); A is the effective cross-sectional area of the cartilage sample for permeation fluid flow (unit: m^2^); ΔP denotes the pressure gradient driving the flow of permeation fluid (unit: Pa); μ represents the dynamic viscosity of the fluid (unit: Pas); and L is the thickness of the cartilage sample (unit: m).

Equation 1 can be rearranged as:

$$k=\frac{Q\;*\;L\;*\;\mu }{A\left(P_{1}-P_{2}\right)}$$ (2)

In the formula, P_1_ denotes the initial pressure of the permeation fluid before flowing through the cartilage sample (Pa), and P_2_ represents the pressure of the permeation fluid after flowing through the cartilage sample (Pa). Additionally, the relationship between force and pressure is expressed as:

$$F=A\;*\;\left(P_{1}-P_{2}\right)$$ (3)

In this study, pressure was only applied to the surface of the cartilage sample, P_2_ = 0. By combining Equations 2, 3, the permeability formula of articular cartilage can be derived as:

$$k=\frac{Q\;*\;L\;*\;\mu }{F}$$ (4)

In the formula, F (N) denotes the hydrostatic pressure applied to the cartilage sample.

Additionally, the volume Q of permeation fluid flowing through the cartilage sample per unit time is expressed as:

$$Q=\frac{d\;*\;\pi \;*\;R^{2}}{t}$$ (5)

In the formula, d denotes the distance of liquid level movement in the permeation tube (m), π is the circular constant (taken as 3.14), and R represents the inner radius of the permeation tube (m). Combining Equations 4, 5 yields:

$$k=\frac{d\;*\;\pi \;*\;R^{2}\;*\;L\;*\;\mu }{\text{tF}}$$ (6)

In conclusion, all the parameters in Formula 6 can be obtained through experiments.

### 2.5 Stability of permeability measurement

#### 2.5.1 Repeatability

For repeatability verification, the core scheme of “single operator + fixed experimental conditions” was adopted. The specific implementation is as follows: First, a systematically trained experimenter conducted the experiments in the same experimental environment, strictly fixing all key variables including the same permeation fluid, cartilage samples with consistent specifications, and the same pressure load (4 N). This ensured that the steps, sequence, and parameters of each operation were completely consistent, with at least six samples measured in each group. To further validate the experimental repeatability, cartilage permeability was detected under the condition of only changing the mechanical load (8 N, 16 N), and the consistency of the results obtained under each mechanical load was verified.

After the measurement, the original permeability data of all parallel cartilage samples and independent groups were collected. The results were compared by calculating the mean $\bar{x}$ , standard deviation (SD), and Coefficient of Variation (CV) of the core indicators.

$$CV=\frac{\text{SD}}{\bar{x}}\;*\;100\%$$ (7)

In Formula 7, if the CV value is ≤ 10%, the repeatability of the determination method is good.

#### 2.5.2 Reproducibility

The reproducibility verification was designed with the core of “multiple operators + unified experimental conditions” to ensure the generalizability of the verification results. Specifically, three experimenters with equivalent experimental operation qualifications jointly familiarized themselves with and strictly followed the uniformly formulated standardized experimental protocol. All operators conducted experiments using cartilage samples of the same specifications, the same batch of permeation fluid, and the same type of instruments calibrated uniformly. The experimental sequence was randomized to avoid systematic errors, and each operator independently completed permeability measurement of six cartilage samples.

After the measurement, the original permeability data of all parallel cartilage samples and independent groups were collected. The results were compared by calculating the mean $\bar{x}$ , standard deviation (SD), and Coefficient of Variation (CV) of the core indicators. If the CV value was ≤10%, and the data of each group showed no significant difference (*P* > 0.05) after Analysis of Variance (ANOVA), the method was deemed to have good reproducibility.

### 2.6 Safranin-O-fast green staining

Cartilage tissues were fixed in 4% paraformaldehyde solution for 24 h, followed by routine paraffin embedding. 5 μm-thick sections were cut to prepare tissue slices, which were placed on a slide warmer for 2 h. Subsequently, the paraffin sections were sequentially immersed in xylene (20 min × 2), absolute ethanol (5 min × 2), 95% ethanol (5 min × 2), and distilled water (5 min × 2) for dewaxing and rehydration.

The sections were immersed in Fast Green staining solution for 5 min, then in 1% acetic acid for 10 s, followed by rinsing with tap water for 5 min. After the slides were air-dried, they were immersed in Safranin-O staining solution for 2 min, and then in distilled water for 5 min to remove excess stain. The sections were sequentially dehydrated by immersion in 95% ethanol (5 s × 2), absolute ethanol (5 s × 2), and xylene (30 s × 2). After air-drying, the slides were mounted with neutral balsam. Once fully dried, the slides were scanned for imaging.

### 2.7 Statistical analysis

All experimental data were expressed as “mean ± standard deviation (mean ± SD)”. Statistical analysis was performed using GraphPad Prism 9.5 software. Intergroup differences were tested by one-way analysis of variance (one-way ANOVA), followed by post hoc multiple comparisons using Fisher’s least significant difference (LSD) method. The significance level for all comparisons was set at *P* < 0.05.

## 3 Results

### 3.1 Friction force measurement and seal integrity testing

#### 3.1.1 Friction force measurement

The inner wall of the permeation tube was in close contact with the piston at the lower end of the push rod. Consequently, frictional force was generated when the push rod moved within the permeation tube, which reduced the set pressure value transmitted to the surface of the osteochondral sample. To quantify the magnitude of this frictional force, the force balance theory for objects moving at a constant velocity on a horizontal surface was adopted: when an object is pushed to move at a constant velocity, the applied thrust equals the frictional force between the object and the surface.

**FIGURE 6:**
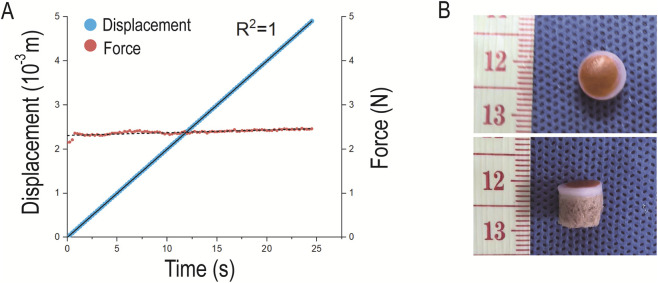
Friction Force Measurement and Seal Integrity Testing. **(A)** Linear fitting of the displacement distance of the push rod piston relative to the permeation tube and the recorded real-time force; **(B)** Top view and side view of the Eosin Y-stained cartilage surface.

Therefore, the lower outlet of the permeation tube was left unsealed, and the central control unit was set to drive the push rod to displace by 5 × 10^-3^ m with an initial displacement of 0 m. The stage then moved upward to compress the push rod, and within the set displacement range, the real-time displacement of the stage was monitored and recorded by the central control unit. As shown in Figure 6A, fitting of the displacement-time curve yielded a linear equation with *R* ^2^ = 1, indicating that the stage moved at a constant velocity—i.e., the piston of the push rod moved uniformly within the permeation tube. The real-time force recorded by the central control unit was 2.5 N, which equals the frictional force between the piston and the inner wall of the permeation tube. Based on this result, the set pressure loads in the subsequent experiments were all adjusted values obtained by subtracting the frictional force (2.5 N) from the initially set values.

#### 3.1.2 Seal integrity testing

The permeability of cartilage was tested using eosin dye as the penetrating fluid. After the test, no trace of eosin dye was observed on the surface of the cartilage in contact with the rubber ring, indicating that the sealing between the cartilage and the rubber ring was good (Figure 6B).

### 3.2 Stability of permeability measurement

#### 3.2.1 Repeatability

**FIGURE 7:**
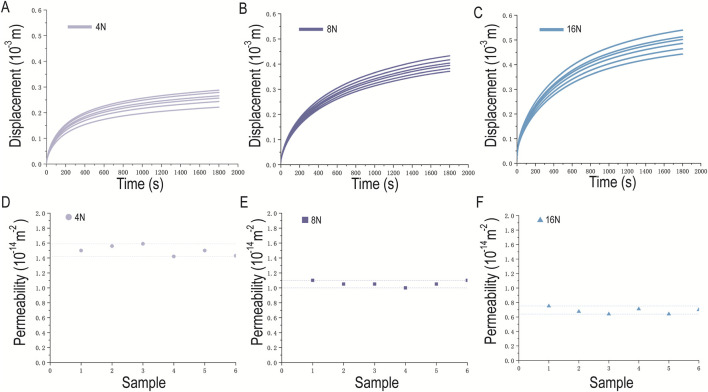
Displacement of the Permeation Fluid Level and Permeability of Osteochondral Samples Under Different Pressure Loads. **(A)** Displacement of the permeation fluid level under 4 N pressure load; **(B)** Displacement of the permeation fluid level under 8 N pressure load; **(C)** Displacement of the permeation fluid level under 16 N pressure load; **(D)** Permeability of each osteochondral sample under 4 N pressure load; **(E)** Permeability of each osteochondral sample under 8 N pressure load; **(F)** Permeability of each osteochondral sample under 16 N pressure load.

A single researcher performed permeability measurements on osteochondral plugs of consistent specifications (n = 6) under an applied pressure load of 4 N with a loading duration of 1800 s. The displacement sensor of the mechanical loading instrument accurately recorded the real-time displacement of the permeation fluid level under the pressure load (Figure 7A). To further validate repeatability, the same researcher conducted permeability measurements on osteochondral plugs of the same specifications under modified pressure loads (8 N, 16 N) while keeping other conditions unchanged (Figures 7B,C). During the 1800-s measurement period, the displacement of the permeation fluid increased with increasing applied pressure load.

The permeability of each osteochondral sample under each pressure load is presented in Table 1. The permeability values measured under each load fluctuated within a narrow range (Figures 7D–F). The mean permeability of the samples was 1.50 × 10^−17^ m^2^ at 4 N, 1.05 × 10^−17^ m^2^ at 8 N, and 0.67 × 10^−17^ m^2^ at 16 N (Table 2). The coefficients of variation (CV) under each pressure load were 4.50%, 3.00%, and 6.40%, respectively, all less than 10.0%. These results indicate that the permeability measurement method exhibits excellent repeatability.

**TABLE 1:** Permeability of cartilage samples.

Sample k (10^−17^ m^2^) Force (N)	1	2	3	4	5	6
4	1.50	1.56	1.59	1.42	1.50	1.43
8	1.10	1.05	1.05	1.00	1.05	1.10
16	0.75	0.68	0.64	0.71	0.64	0.70

**TABLE 2:** Average permeability and CV values of cartilage samples in each group under different pressure loads.

Parameter Force (N)	Mean value ( $\bar{x}$ )	Standard deviation (SD)	Coefficient of variation (CV)
4	1.50	0.068	4.5%
8	1.05	0.032	3.0%
16	0.67	0.043	6.4%

#### 3.2.2 Reproducibility

**FIGURE 8:**
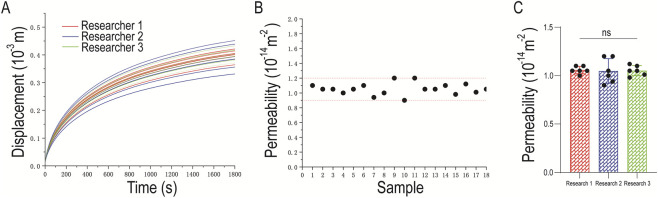
Permeability Measurement of Osteochondral Samples Performed by Three Researchers. **(A)** Displacement of the permeation fluid level during permeability measurement of osteochondral samples by three researchers; **(B)** Permeability of each individual osteochondral sample; **(C)** Statistical chart of osteochondral permeability measured by three researchers; one-way ANOVA revealed no significant difference among groups (all *P* > 0.05).

Three researchers performed permeability measurements on osteochondral plugs of the same specifications. The displacement sensor of the mechanical loading instrument accurately recorded the real-time displacement of the permeation fluid level under the applied pressure load (Figure 8A). The permeability of each individual osteochondral sample is presented in Table 3, and all permeability values fluctuated within a reasonable range (Figure 8B).

**TABLE 3:** Permeability of cartilage samples measured by three researchers.

Sample k (10^−17^ m^2^) Research	1	2	3	4	5	6
Researcher 1	1.10	1.05	1.05	1.00	1.05	1.10
Researcher 2	0.94	1.00	1.20	0.90	1.20	1.05
Researcher 3	1.05	1.10	0.98	1.12	1.01	1.05

The mean permeability of the samples measured by the three researchers was 1.05 × 10^−17^ m^2^ with a standard deviation (SD) of 0.077945, resulting in a coefficient of variation (CV) of 7.4%. Additionally, one-way analysis of variance (ANOVA) revealed no statistically significant difference in permeability among the three researchers (*P* = 0.9776 > 0.05) (Table 4, Figure 8C). These results collectively indicate that the permeability measurement method exhibits excellent reproducibility.

**TABLE 4:** One-way analysis of variance results of cartilage permeability measured by three researchers.

Group	n	Permeability k (10^−17^ m^2^) $\bar{x}\pm SD$
Researcher 1	6	1.0583 ± 0.0377
Researcher 2	6	1.0483 ± 0.1281
Researcher 3	6	1.0517 ± 0.0527
*F*	​	0.02264
*P*	​	0.9776

### 3.3 Application of the cartilage permeability measurement device

#### 3.3.1 Permeability of porcine knee joint cartilage to clinically used substances

**FIGURE 9:**
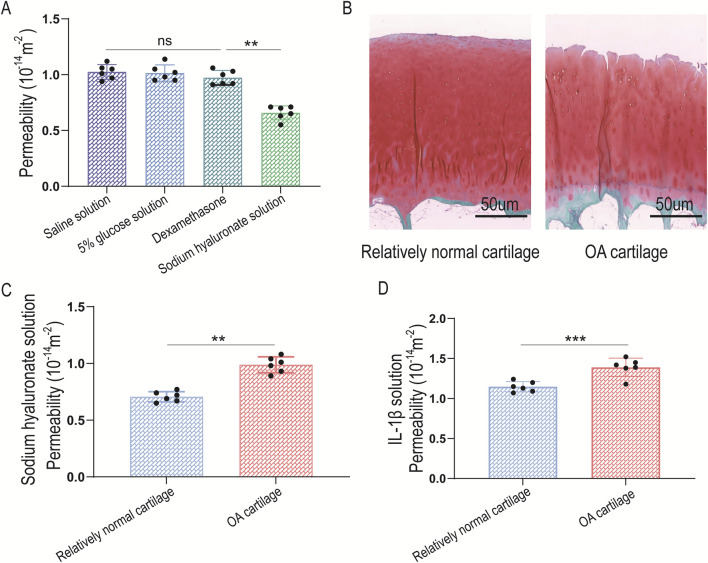
Permeability detection of porcine and human knee joint cartilage. **(A)** Statistical analysis of the permeability of porcine knee cartilage to clinically common solutes; **(B)** Safranin-O-Fast Green staining of human knee joint cartilage; **(C)** Permeability of relatively normal human knee joint cartilage and osteoarthritic (OA) cartilage to sodium hyaluronate injection; **(D)** Permeability of relatively normal human knee joint cartilage and osteoarthritic (OA) cartilage to IL-1β solution.

Normal saline (μ = 1 mPa·s), 5% glucose solution (μ = 1.2 mPa·s), sodium hyaluronate injection (μ = 27 mPa·s), and dexamethasone injection (μ = 1.2 mPa·s) were selected as clinically used solutes. The average permeability (k) of cartilage to these substances was measured as 1.03 × 10^−17^ m^2^, 1.02 × 10^−17^ m^2^, 0.97 × 10^−17^ m^2^, and 0.64 × 10^−17^ m^2^, respectively (Table 5). Sodium hyaluronate injection exhibited a higher dynamic viscosity than the other substances, and the cartilage permeability to it was significantly lower (*P* = 0.0083 < 0.01) (Figure 9A).

**TABLE 5:** Permeability of pig knee joint cartilage to common clinical substances.

Sample k (10^−17^ m^2^) Solution	1	2	3	4	5	6	$\bar{x}$
Saline solution	1.01	0.94	0.97	1.05	1.12	1.06	1.03
5% glucose solution	0.95	0.95	1.02	1.14	1.05	0.98	1.02
Dexamethasone	1.03	0.92	0.91	1.00	1.06	0.92	0.97
Sodium hyaluronate solution	0.63	0.55	0.71	0.65	0.61	0.70	0.64

#### 3.3.2 Permeability of degenerated human knee joint cartilage to sodium hyaluronate injection and Interleukin-1β solution

Relatively normal human knee joint cartilage and degenerated cartilage from patients with osteoarthritis (OA) were selected (Figure 9B). The permeability of these cartilages to a clinical therapeutic drug (sodium hyaluronate injection) and an inflammatory factor (IL-1β) solution was measured.

For sodium hyaluronate injection (Table 6), the permeability of relatively normal cartilage and OA-degenerated cartilage was 0.71 × 10^−17^ m^2^ and 0.99 × 10^−17^ m^2^, respectively. For IL-1β solution (Table 7), the permeability was 1.15 × 10^−17^ m^2^ and 1.39 × 10^−17^ m^2^, respectively (Tables 6, 7). When cartilage degeneration occurs, the permeability to both therapeutic substances and inflammatory factors increased significantly, with statistically significant differences (*P* = 0.0011 < 0.01, *P* = 0.0046 < 0.01) (Figures 9C,D).

**TABLE 6:** Permeability of human knee joint cartilage to sodium hyaluronate Injection.

Sample k (10^−17^ m^2^) Cartilage	1	2	3	4	5	6	$\bar{x}$
Relatively normal cartilage	0.72	0.69	0.65	0.74	0.77	0.67	0.71
OA cartilage	0.98	1.08	0.79	1.01	1.04	0.93	0.97

**TABLE 7:** Permeability of human knee joint cartilage to interleukin-1 β solution.

Sample k (10^−17^ m^2^) Cartilage	1	2	3	4	5	6	$\bar{x}$
Relatively normal cartilage	1.13	1.09	1.24	1.07	1.15	1.20	1.15
OA cartilage	1.42	1.38	1.39	1.45	1.52	1.18	1.39

## 4 Discussion

The core objective of this study is to address key limitations of existing articular cartilage permeability detection technologies, including detachment from the physiological environment, insufficient quantification accuracy, and poor sealing performance. A precise, reliable, and in vivo-relevant detection device and method were developed to provide technical support for elucidating cartilage physiological functions, investigating the mechanisms of degenerative diseases, and evaluating tissue-engineered cartilage.

To achieve this goal, based on the intra-articular convective transport mechanism and Darcy’s Law, this study designed a detection device with a modular structure that corely replicates the “synovial fluid–cartilage–subchondral bone” functional unit. A sealing system was constructed using a quartz glass permeation tube and a high-strength stainless steel push rod. Compressibility-induced interference was eliminated by replacing gas pressurization with liquid pressurization, and dual high-precision measurement of pressure and displacement was achieved by combining the ElectroForce 3200 mechanical loading system with high-resolution displacement sensors. Using knee joint osteochondral plugs as samples, the experiment proceeded through sample preparation, sealed installation, mechanical loading, and data collection, with quantitative calculation ultimately completed via the derived permeability formula. Additionally, verification of friction force, sealing performance, repeatability, and reproducibility was conducted.

To reproduce the closed environment of the *in vivo* joint cavity and the essence of convective transport, the device design adopted a structure where a rubber ring is embedded in the groove at the lower end of the permeation tube. This structure not only utilizes the elasticity of rubber to adapt to irregular cartilage surfaces and replicate the sealing characteristics of the joint cavity but also avoids pressure fluctuations caused by gas compressibility by replacing gas pressurization with liquid pressurization. Meanwhile, based on the physical principle that thrust equals friction force when an object moves at a constant velocity, a friction force quantification protocol of “sample-free uniform displacement” was designed. This mechanism design directly yielded clear results: no leakage of eosin dye was observed in the sealing test, and the friction force was quantified as 2.5 N. The significance of this outcome lies not only in “addressing the long-standing drawbacks of traditional detection” but also in the rationality of the underlying mechanism. Precisely because the sealing structure is bionically adapted to the *in vivo* closed environment and liquid pressurization is consistent with the physiological mechanism of cartilage convective permeation, the unidirectionality of the permeation pathway and the accuracy of pressure transmission are ensured. This provides a fundamental guarantee for the precision of subsequent permeability calculations and endows the device with the core advantage of “being relevant to *in vivo* reality.”

The repeatability and reproducibility of the detection method essentially rely on the triple mechanistic support of “device structural stability, theoretical model adaptability, and operational procedure standardization” (the second core mechanism). In this study, the modular device adopted low-deformation quartz glass and high-strength stainless steel materials to ensure no interference from structural deformation during pressure loading. The permeability formula derived from Darcy’s Law (Formula 6) accurately correlates the quantitative relationship between pressure, displacement, sample parameters, and permeability, avoiding theoretical deviations. Meanwhile, a standardized protocol was formulated featuring unified sample specifications, unified instrument calibration, and unified operational steps.

Under the guarantee of this mechanism, clear experimental results were obtained: In the repeatability tests, the average cartilage permeability under pressures of 4 N, 8 N, and 16 N was 1.50 × 10^−17^ m^2^, 1.05 × 10^−17^ m^2^, and 0.67 × 10^−17^ m^2^, respectively, with all coefficients of variation (CV) ≤ 6.4%. In the reproducibility tests, the average permeability measured by three operators was 1.05 × 10^−17^ m^2^ with a CV of 7.4%, and one-way analysis of variance (ANOVA) showed no significant intergroup difference (*P* = 0.9776 > 0.05). The significance of this result lies in the fact that its reliability is not accidental, but an inevitable outcome of the synergistic effect of the device structure, theoretical model, and operational procedures. This not only confirms the stability of the method under fixed conditions and its universality among different operators, but also endows it with the potential for technical standardization and cross-laboratory promotion, providing a reliable quantitative tool for subsequent cartilage-related research.

“Regulation of cartilage permeability by its viscoelasticity and hierarchical pore network” is another core mechanism of this study. Articular cartilage is composed of type II collagen fibers and glycosaminoglycans (GAGs), forming hierarchical pore channels ranging from 4–6 nm to 50–100 nm (Guo et al., 2025). Hydrostatic pressure induced by *in vivo* joint movement causes physiological compression, which in turn affects pore structure and fluid transport efficiency (Ebrahimi et al., 2019; Greene et al., 2008). Based on this physiological mechanism, in the repeatability tests, and a key result was obtained: pressure load is negatively correlated with permeability—higher pressure leads to lower permeability. This result is consistent with the findings of Michael E Stender’s research (Stender et al., 2017). The significance of this result goes far beyond “discovering a regularity”; its underlying mechanism explains why excessive *in vivo* joint movement may lead to cartilage damage. When pressure load is applied, cartilage undergoes physiological compression: On one hand, higher pressure significantly reduces the porosity of the cartilage matrix, narrows the gaps between collagen fibrils and GAGs, and thus constricts the effective channels for fluid transport, hindering the trans-matrix transport of permeants (Raya, 2015). On the other hand, the “creep effect” of viscoelastic materials causes cartilage deformation to gradually stabilize during sustained pressure loading, but the proportion of irreversible compression deformation increases under high pressure, further reducing pore connectivity (Linus et al., 2024). Although the pressure gradient driving permeant flow increases under high pressure, leading to a higher total liquid level displacement, permeability—as a parameter characterizing the intrinsic transport capacity of cartilage—depends primarily on the matrix pore structure and fixed charge density. The destructive effect of pressure-induced cartilage compression on the pore structure dominates, ultimately resulting in decreased permeability with increasing pressure. This finding reveals the nonlinear relationship between pressure load and cartilage permeability, providing new experimental evidence for an in-depth understanding of the regulatory mechanism of *in vivo* joint movement on cartilage substance exchange, this result is consistent with the findings of James M Fick‘s research (Fick et al., 2010).

This study is not without limitations. In the experiments, knee joint cartilage from 6-month-old Landrace pigs was used as samples, which could meet the requirements of device verification. However, there are species differences between porcine and human cartilage in matrix components (e.g., GAG content, collagen fiber arrangement), mechanical properties, and pore structure, which may prevent the direct extrapolation of measurement results to human cartilage (Malda et al., 2012; Taylor et al., 2012). Future studies need to further verify the applicability of this method in human cartilage samples (such as surgically resected pathological cartilage). Additionally, the experimental samples were ex vivo-prepared osteochondral plugs. Although freshness was maintained as much as possible, the *ex vivo* environment can lead to the gradual loss of chondrocyte viability and potential degradation of matrix components (e.g., GAGs), thereby affecting the viscoelasticity and permeability of cartilage. In the future, a low-temperature viability preservation system should be established, or fresh *in vivo* samples should be used for experiments to ensure that the detection results can reflect the physiological state of cartilage. On the other hand, the hydrostatic pressure generated during joint movement is cyclic and dynamically changing (0.1–3 MPa), accompanied by cartilage shear deformation caused by joint flexion and extension (Kazemi et al., 2012; Komala et al., 2025). This study applied a constant pressure load, which cannot simulate the time-varying effects of the *in vivo* dynamic mechanical environment on cartilage permeability. Future work needs to optimize the mechanical loading system to realize the application and regulation of cyclic loads.

Despite certain limitations, the core findings of this study still provide critical support for the articular cartilage field at the theoretical, technical, and practical levels. At the theoretical level, taking the in vivo-dominant convective transport as the core mechanism, this study replicated the “synovial fluid–cartilage–subchondral bone” functional unit and, for the first time, obtained cartilage permeability under pressure loads through precise quantification. This fills the gap that traditional static detection methods cannot reflect the dynamic fluid transport characteristics under physiological mechanical environments, further improving the correlation theory between cartilage permeability and biomechanical functions. At the technical level, this study targetedly addressed the core drawbacks of traditional methods: gas compressibility interference was eliminated via liquid pressurization, the edge leakage problem was solved by the elastic sealing design of rubber rings, and a precise quantification system was established by combining micrometer-level displacement sensors with Darcy’s Law-derived formulas. Verified to have excellent repeatability (CV ≤ 6.4%) and reproducibility (CV = 7.4%, *P* = 0.9776 > 0.05), this study provides a standardized technical tool with both “physiological relevance” and “quantification accuracy” for cartilage permeability detection, breaking the limitations of existing dynamic detection methods such as complex operation and poor result comparability. Meanwhile, the modular device design and standardized operational procedures have laid a foundation for the subsequent development of portable clinical detection equipment. It is expected to become a real-time functional evaluation method for cartilage injury, filling the gap of functional quantitative indicators in the current cartilage injury repair evaluation system and promoting the development of cartilage regeneration technologies and the diagnosis and treatment of degenerative joint diseases.

## 5 Conclusion

Based on the *in vivo* convective transport mechanism and Darcy’s Law, this study successfully developed a modular device for detecting articular cartilage permeability. By replicating the “synovial fluid–cartilage–subchondral bone” functional unit and adopting liquid pressurization combined with an elastic rubber ring sealing design, the device effectively addresses the core limitations of traditional technologies, including pressure fluctuations, edge leakage, and detachment from physiological scenarios.

Featuring both physiological relevance and quantitative precision, this method provides a reliable technical tool for elucidating the physiological mechanisms of articular cartilage. It holds significant implications for improving the cartilage injury repair evaluation system and promoting the clinical translation of cartilage regeneration technologies.

## Data Availability

The original contributions presented in the study are included in the article/supplementary material, further inquiries can be directed to the corresponding authors.
